# Supplementary material for: High-order sensory processing nanocircuit based on coupled VO2 oscillators
Source: Nat Commun. 2024 Feb 24;15:1693. doi: 10.1038/s41467-024-45992-8 (PMC10894221; doi:10.1038/s41467-024-45992-8)
Supplement: Supplementary file 1 — Supplementary Information [file 41467_2024_45992_MOESM1_ESM.pdf]

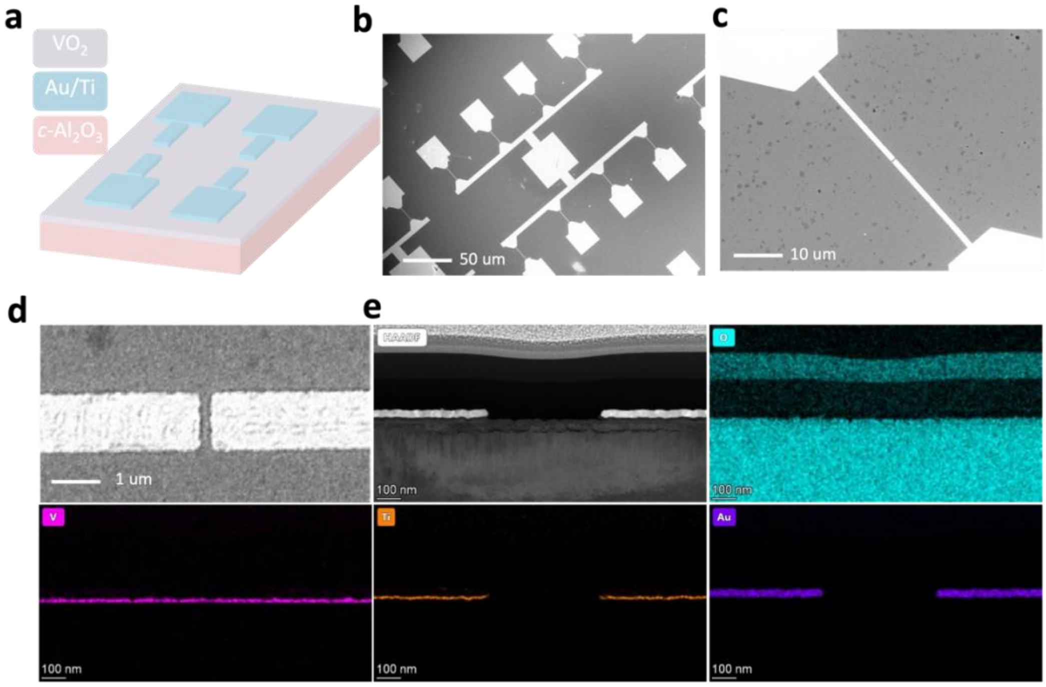

Supplementary Figure 1. Microstructural and compositional characterization of VO2 device. (a) Diagram of a planar VO_2 device's structure. (b-d) SEM mapping of VO_2 devices. (e) Cross-sectional STEM image and corresponding EDS mapping of O, V, Au, Ti elements in the device.

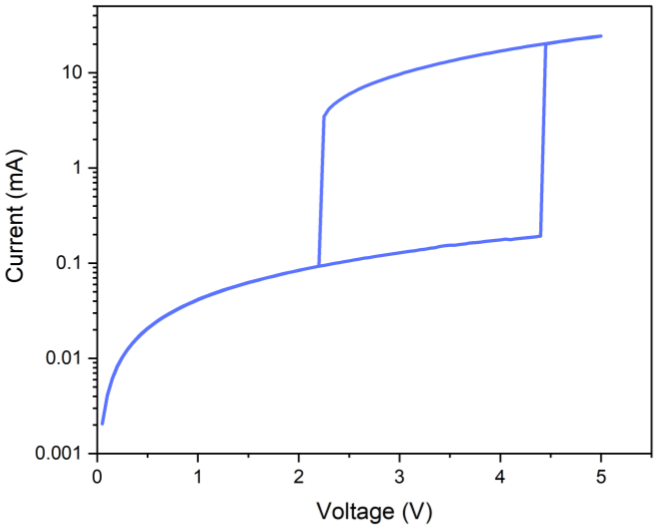

Supplementary Figure 2. Experimental quasi-static voltage scanning of a VO_2 device without compliance current. The maximum current through the device can reach to around 20 mA.

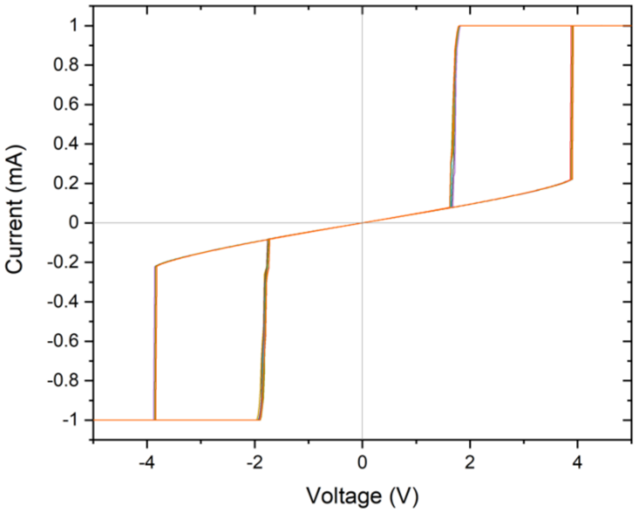

Supplementary Figure 3. Experimental bidirectional quasi-static voltage scanning of a VO_2 device (10 cycles).

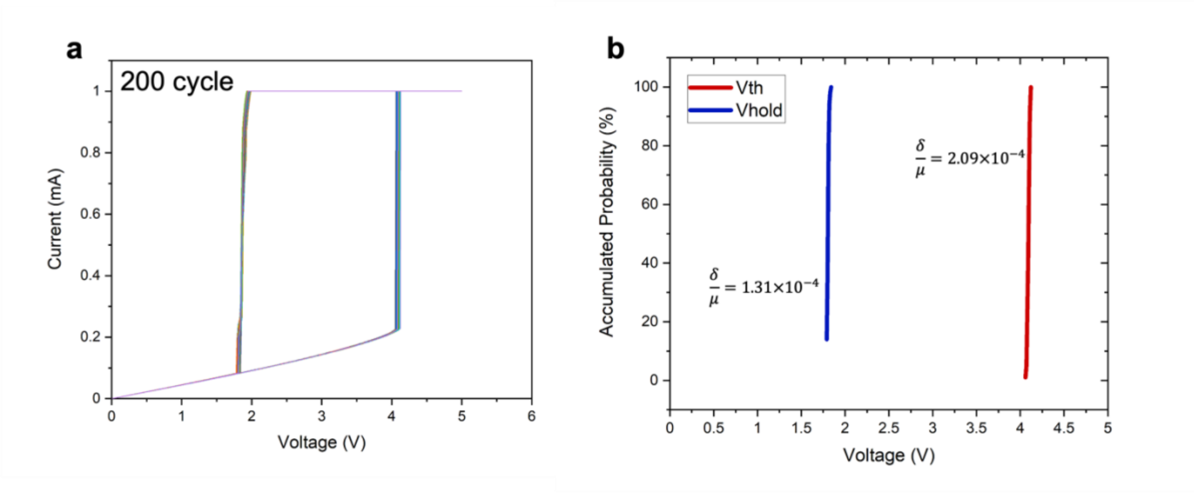

Supplementary Figure 4. Measurement of VO_2 device cycle-to-cycle variation. (a) quasi-static voltage scanning of a VO_2 device (200 cycles). (b) The cycle-to-cycle variation of VO_2 device's threshold voltage and hold voltage.

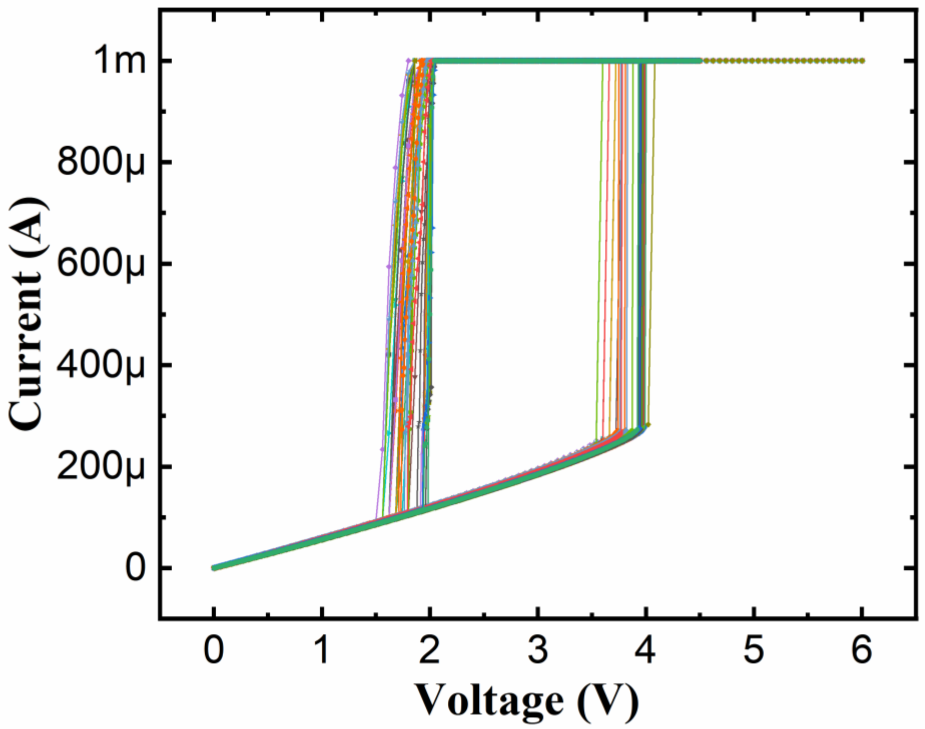

Supplementary Figure 5. Measurement of VO_2 device device-to-device variation in 40 devices.

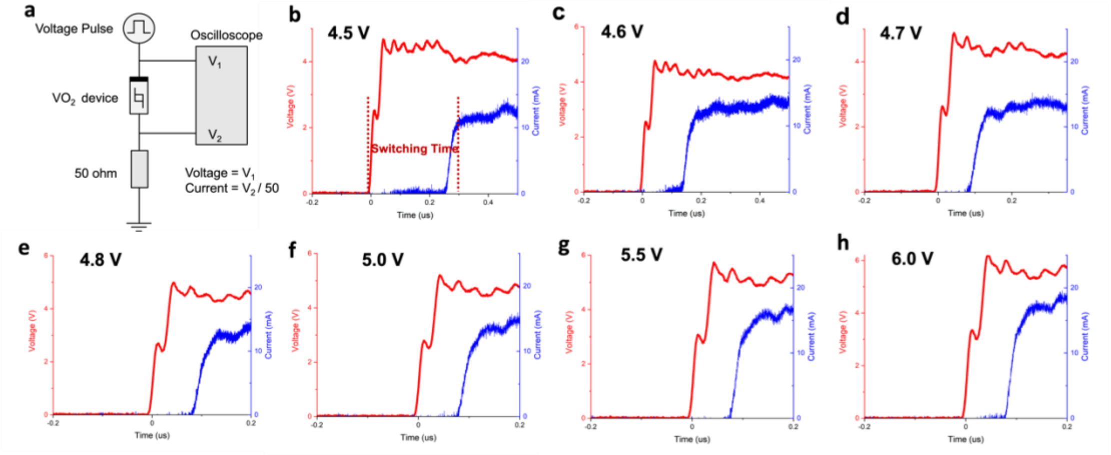

Supplementary Figure 6. Experimental measurements of VO_2 device's switching time from off-state to on-state. (a) The testing circuit diagram (b-h) Time difference between the voltage input and the current output when the VO_2 device switches from off-state to on-state, whose threshold voltage is around 4.5 V.

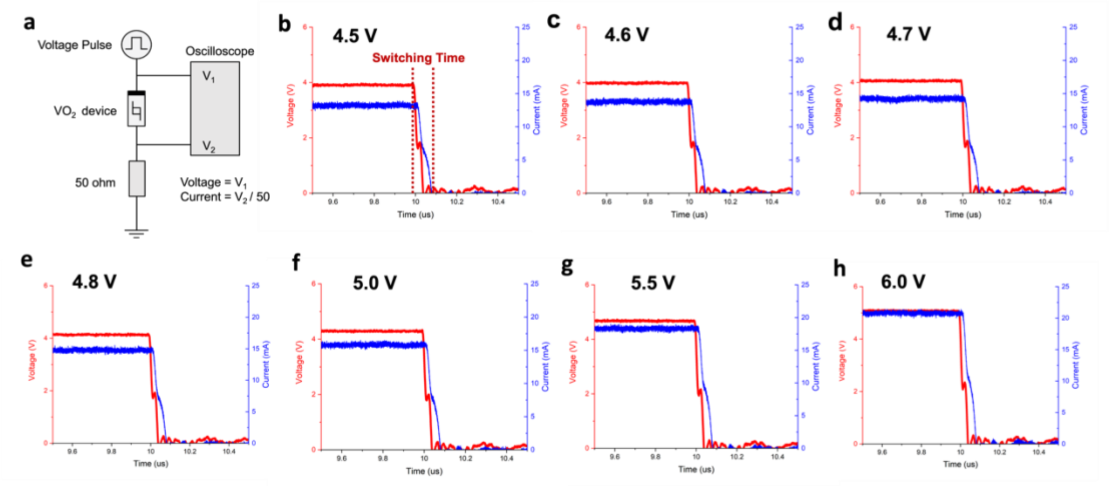

Supplementary Figure 7. Experimental measurements of VO_2 device's switching time from on-state to off-state. (a) The testing circuit diagram (b-h) Time difference between the voltage input and the current output when the VO_2 device switches from on-state to off-state, whose threshold voltage is around 4.5 V.

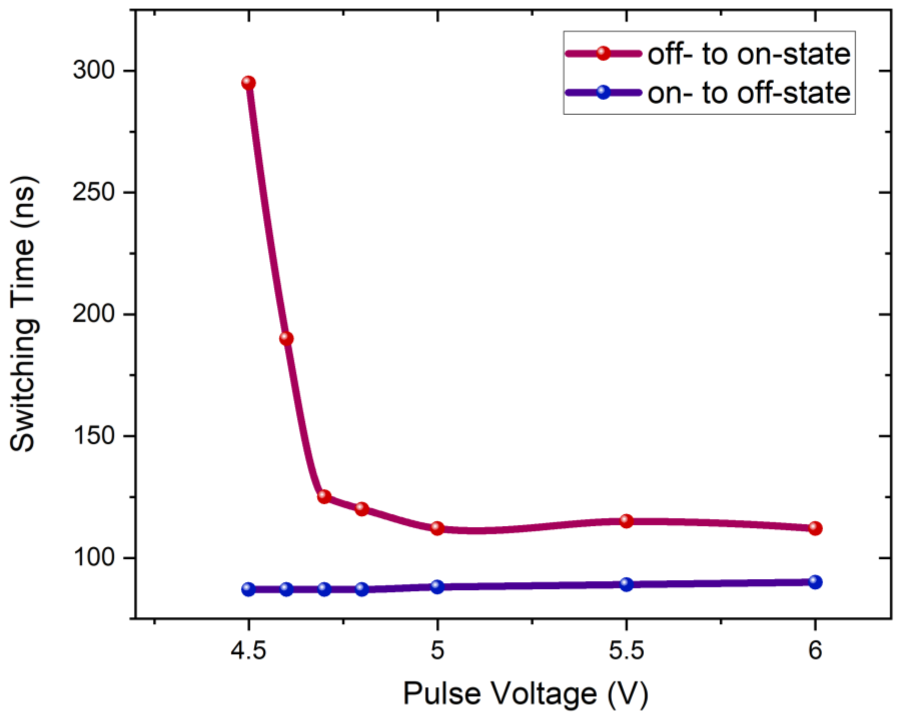

Supplementary Figure 8. Experimental measurements of the relationship between the VO_2 device's switching time and the applied pulse voltage. The switching time limits the maximum frequency around 2.6 MHz.

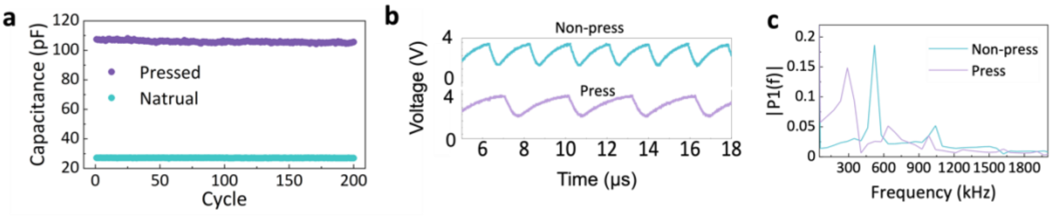

Supplementary Figure 9. The electrical characteristics of the pressure sensor and the corresponding sensory VO_2 oscillator. (a) The capacitance characteristic of the pressure sensor. (b) The typical waveform of the haptic sensory neuron with the pressure sensor. (c) Single-sided amplitude spectrum though FFT of the waveforms in b).

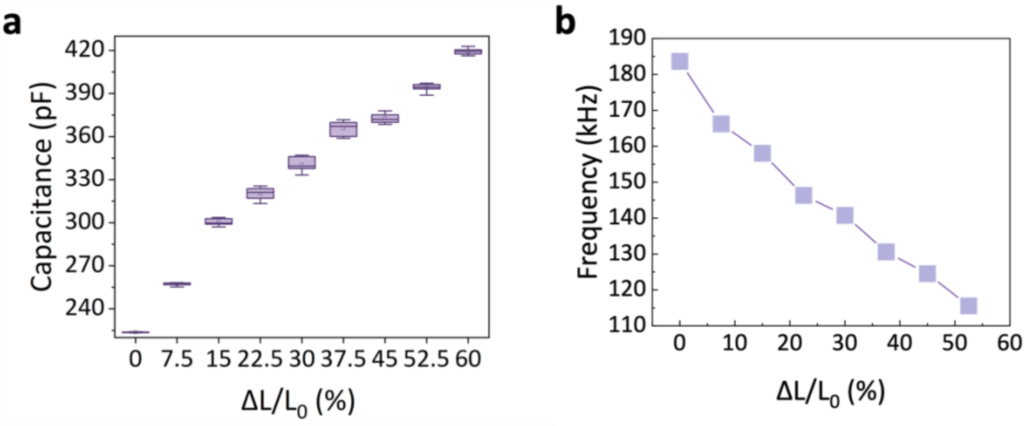

Supplementary Figure 10. The electrical characteristics of the stretching sensor and the corresponding sensory VO_2 oscillator. (a) The capacitance characteristic of the stretching sensor regarding varied stretch lengths ΔL. (b) The relationship between the stretch lengths ΔL and the output frequency.

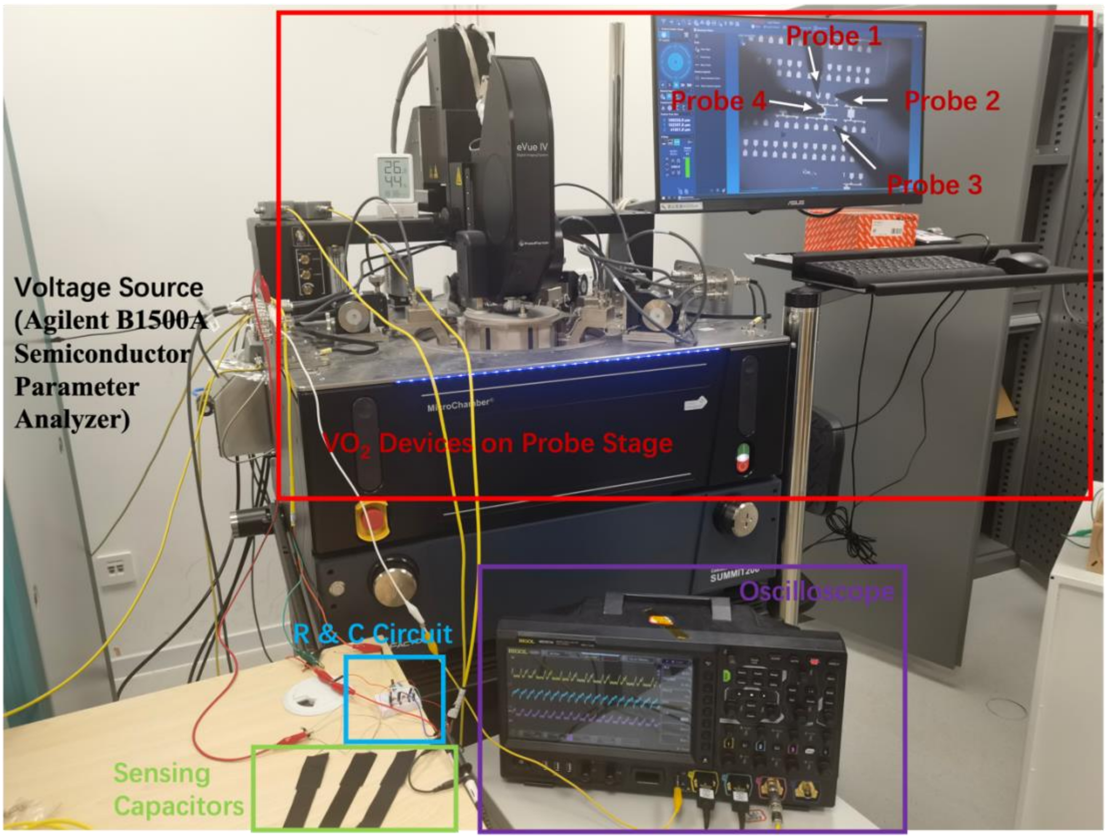

Supplementary Figure 11. A visual representation of the experiment setup, along with block diagrams of the components used.

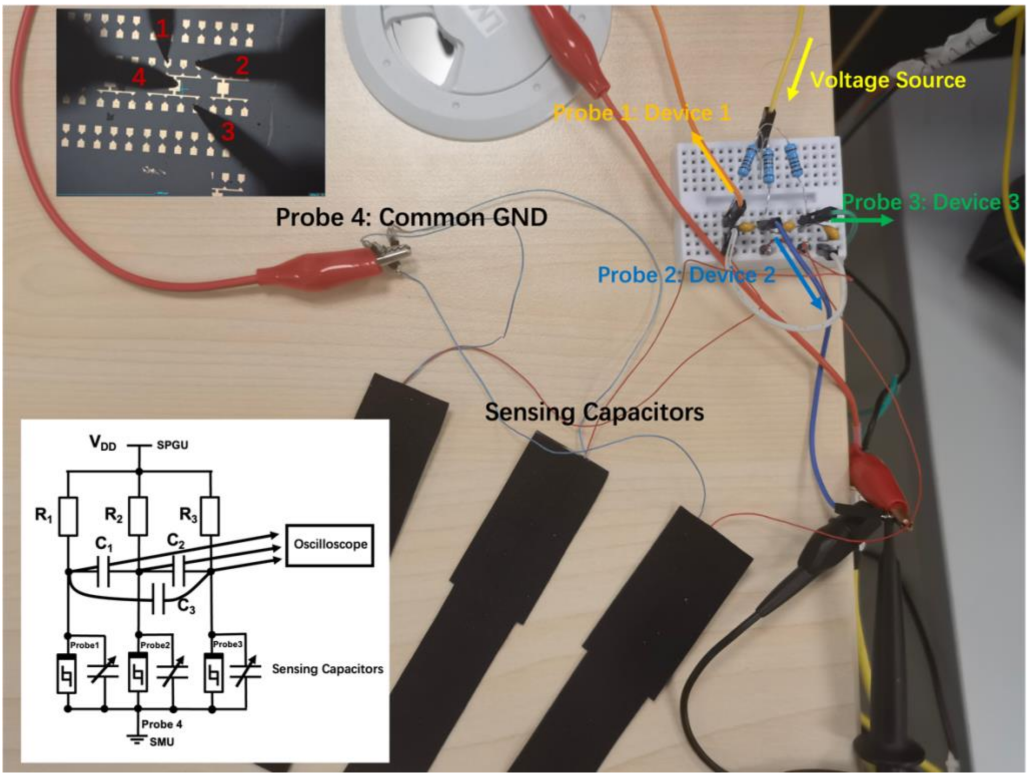

Supplementary Figure 12. A picture of experiment setup and used components with explanatory schematics.

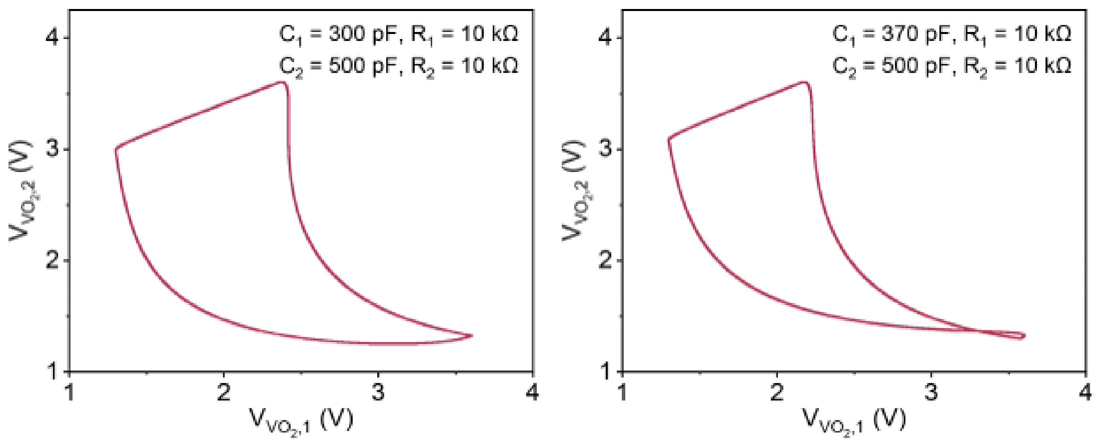

Supplementary Figure 13. The phase relationship of two memristive sensory oscillators coupled by a parallel capacitor in synchronization (in simulation).

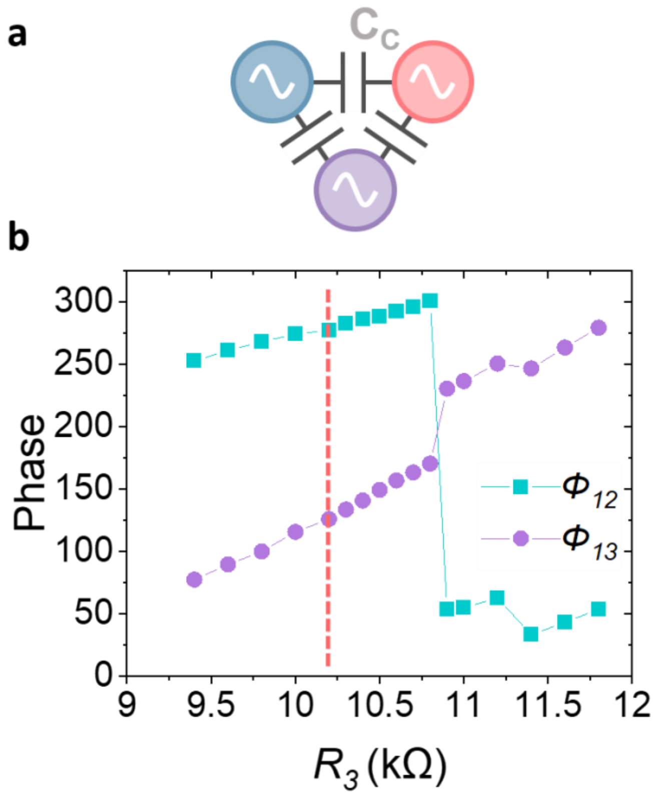

Supplementary Figure 14. Synchronization between three oscillatory neurons through capacitances. (a) Circuit diagram of three coupled oscillatory neurons through capacitances. (b) Phase difference of three coupled oscillators when keeping $f_1 = f_2$ and modulating the phase pattern by f_3 through changing series resistor in oscillators.

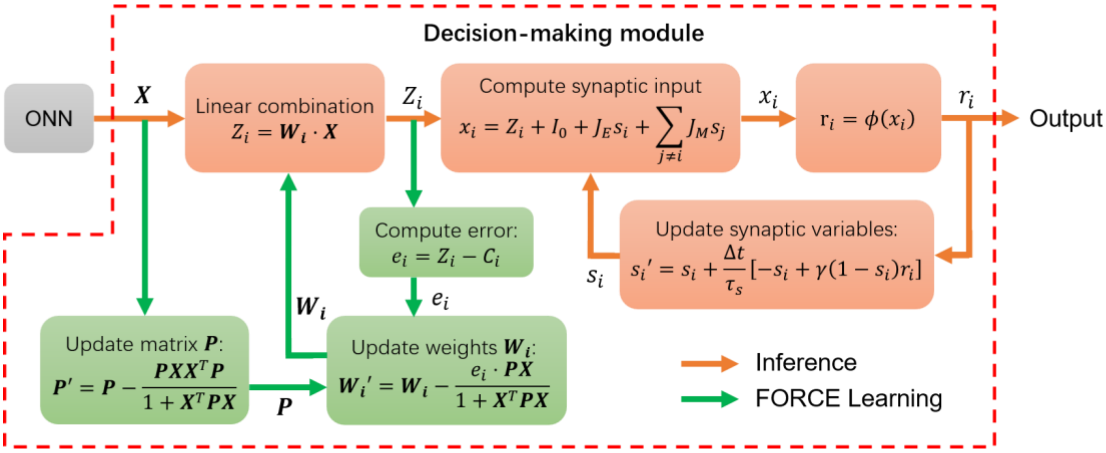

Supplementary Figure 15. Detailed decision-making module and FORCE learning algorithm processing flow in high-level processing to post-processing continuous time signals from ONN. In the inference process, the voltage oscillation signals **X** from the coupled oscillators' first pass through the linear combination and turn to **Z**. The signal **Z** is the input of the decision-making module, computing the synaptic input and computing a nonlinear activation function, turning to **r** as the output in the end. Because all the signals are time-continuous, the slow volatile dynamic of synapses are also included to compute **s,** which can be seen as the synapse current in inter-connection and self-connection. In the Force Learning training process, we set training target output **Z**, and compute the error **e** between target **Z** and actual output **C**. Then using the error function to adjust the weight of the linear combination **W** with an update matrix **P.**

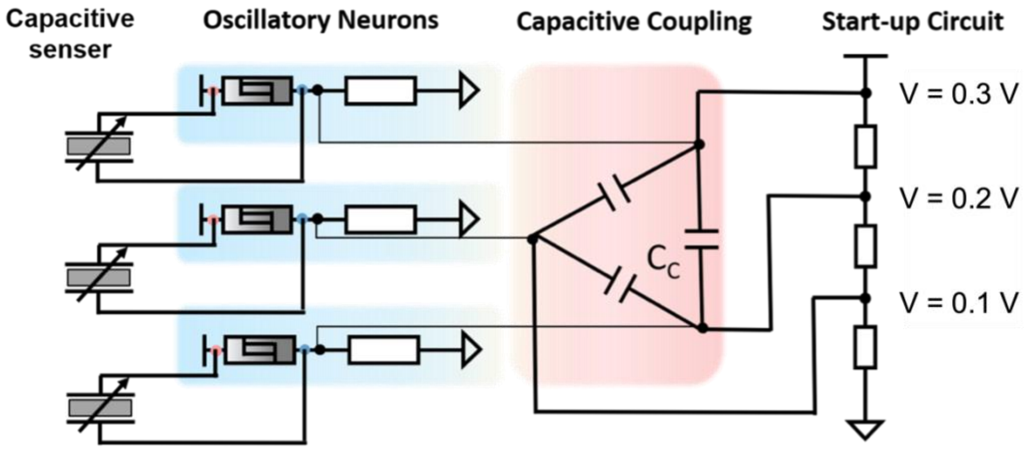

Supplementary Figure 16. Start-up Circuit with ONN of three coupled oscillatory neurons with a capacitive sensor. The Start-up circuit is to make sure the initial phase doesn't affect the phase difference after coupling, keeping the initial state stability of the dynamic computing system.

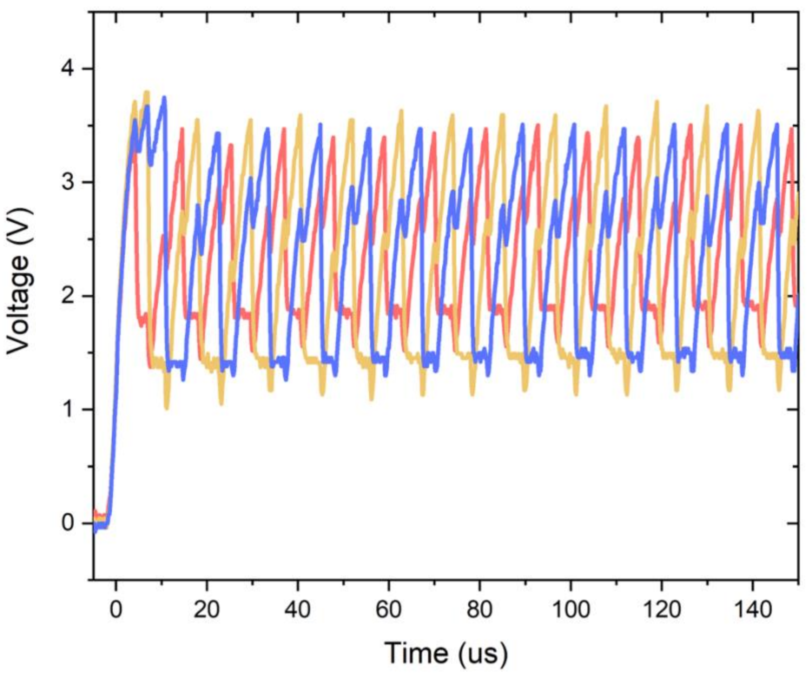

Supplementary Figure 17. The transient coupling process of three coupled sensory VO_2 oscillators in actual experiment. The transient time is less than 20 us.

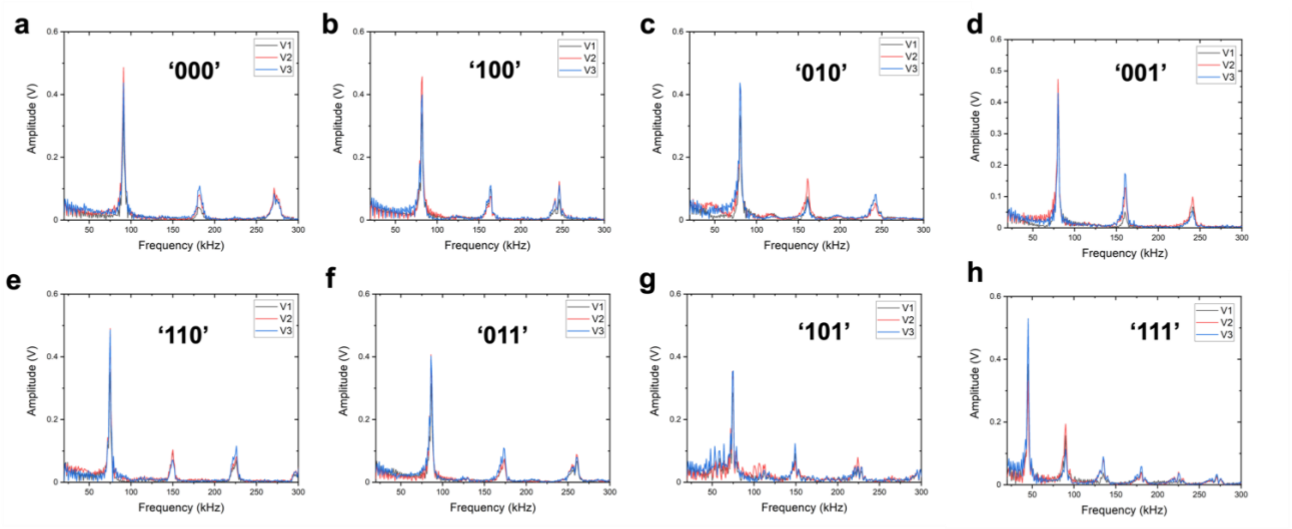

Supplementary Figure 18. The FFT result of three coupled sensory VO_2 oscillators in all eight cases, where '1' and '0' represent the pull/not pull the sensing capacitance. (a) Case '000'. (b) Case '100'. (c) Case '010'. (d) Case '001'. (e) Case '110'. (f) Case '011'. (g) Case '101'. (h). Case '111'.

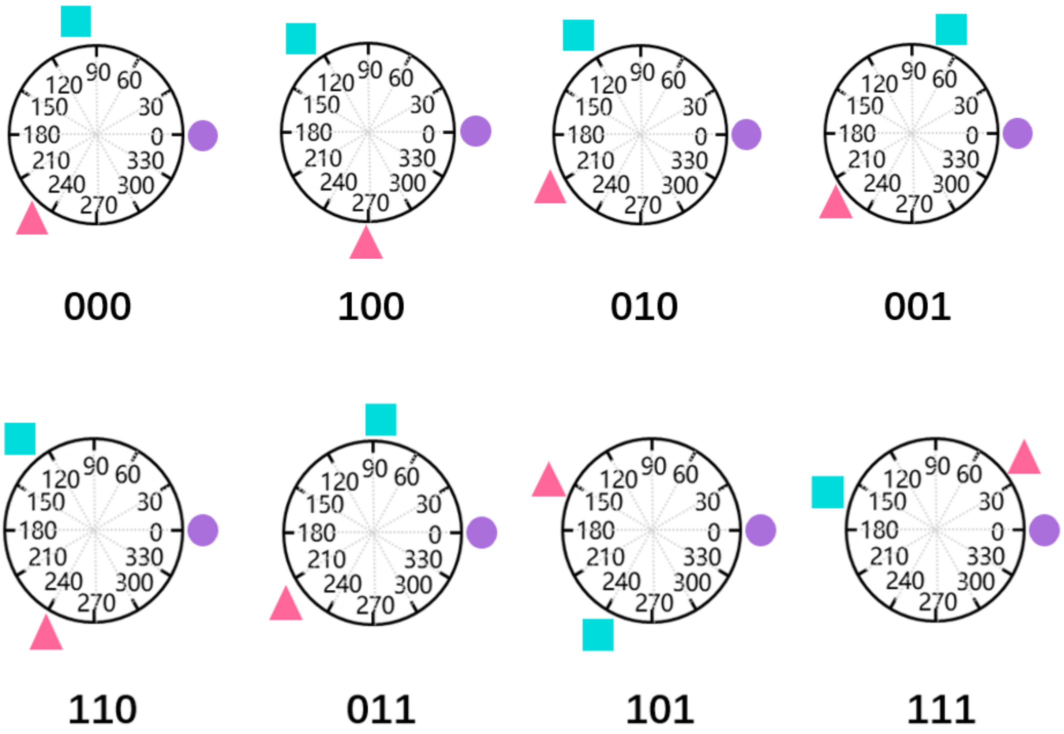

Supplementary Figure 19. Eight phase modes corresponding to eight sensing inputs in a coupled ONN with three VO_2 oscillators. The ‘1’ and ‘0’ represent the pull/not pull the sensing capacitance.

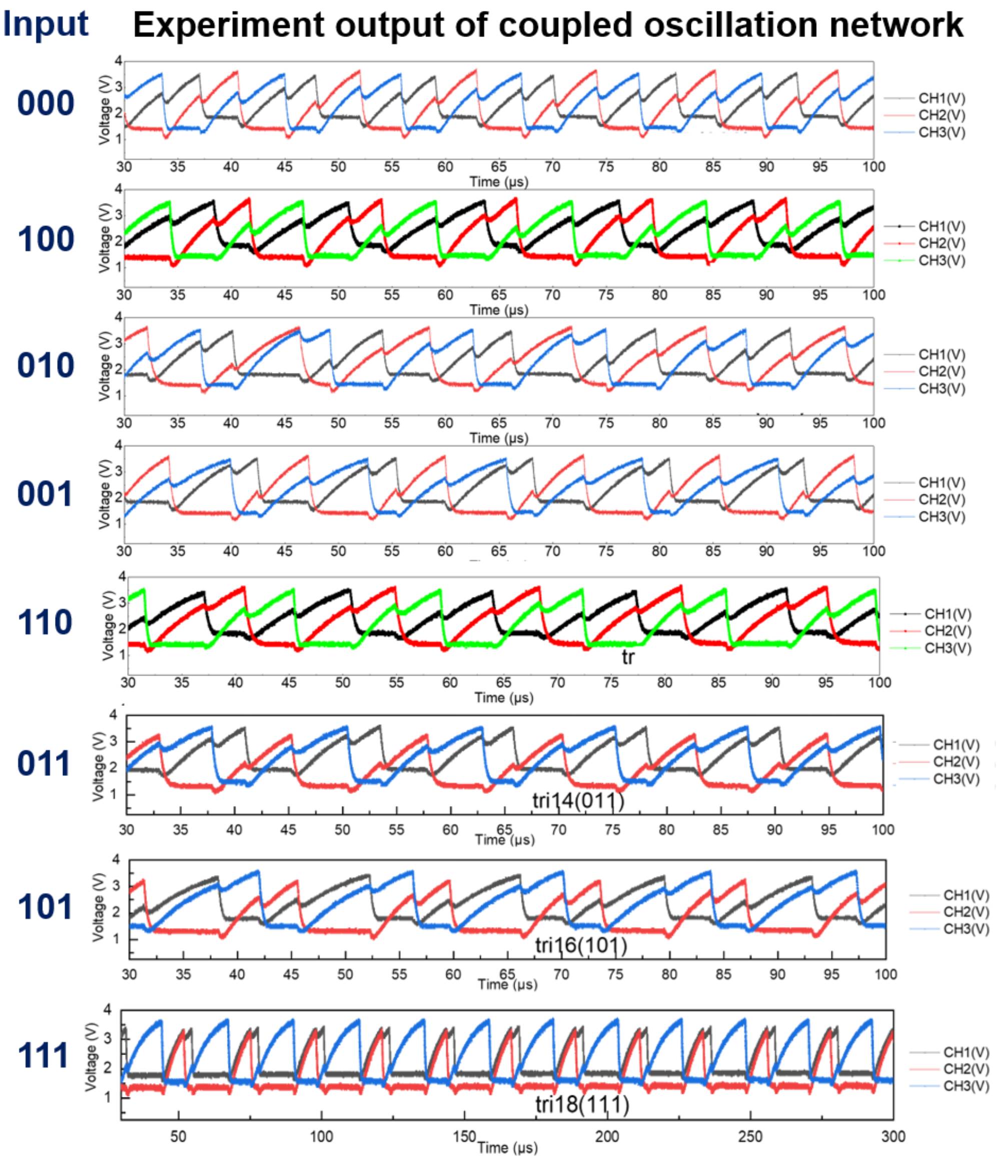

Supplementary Figure 20. The experiment output of coupled oscillation neural network based on three VO_2 oscillators corresponding to eight sensor input combinations. The ‘1’ and ‘0’ represent the pull/not pull the sensing capacitance.

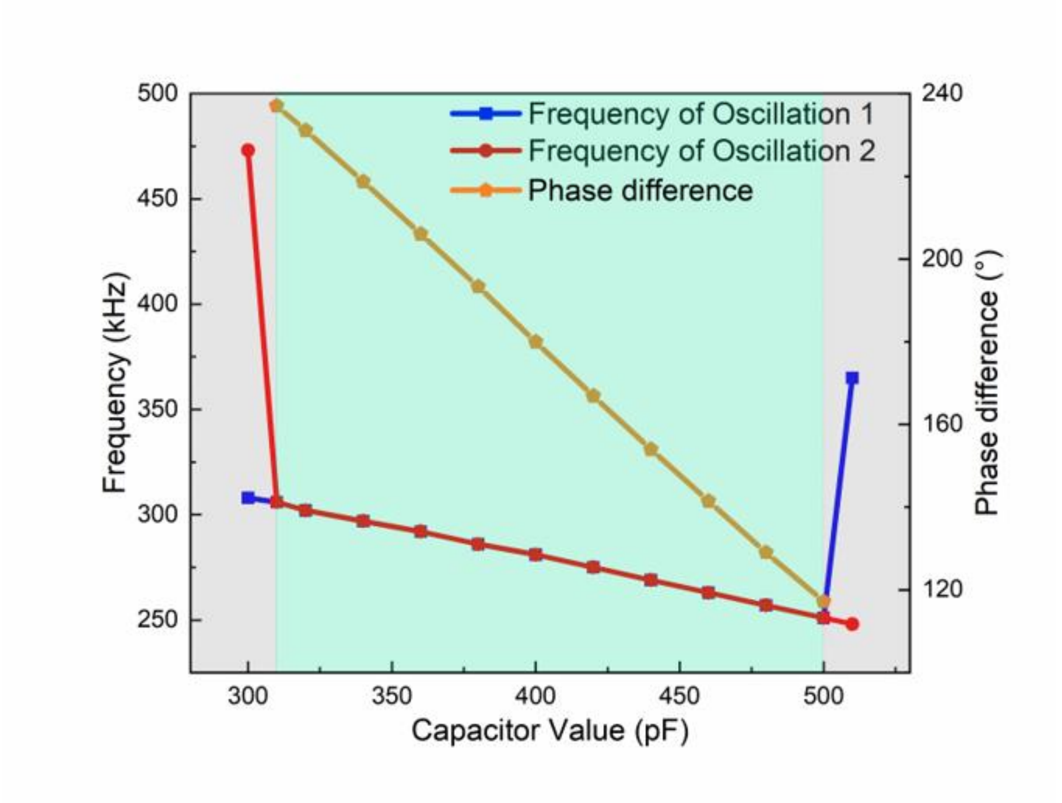

Supplementary Figure 21. Influence of sensing capacitor to two oscillators' coupling. The green background indicates synchronization, while the gray background suggests desynchronization.

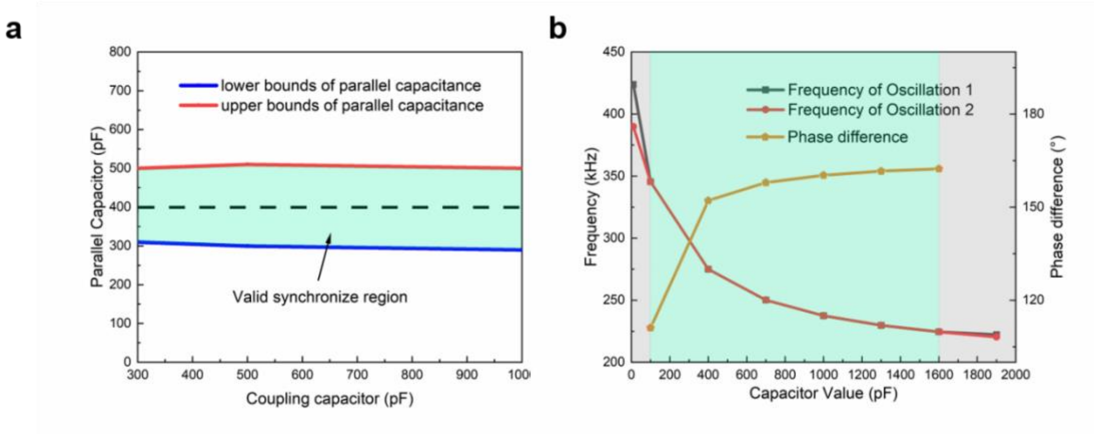

Supplementary Figure 22. Influence of coupling capacitor to two oscillators' coupling without (a) and with (b) device to device variation.

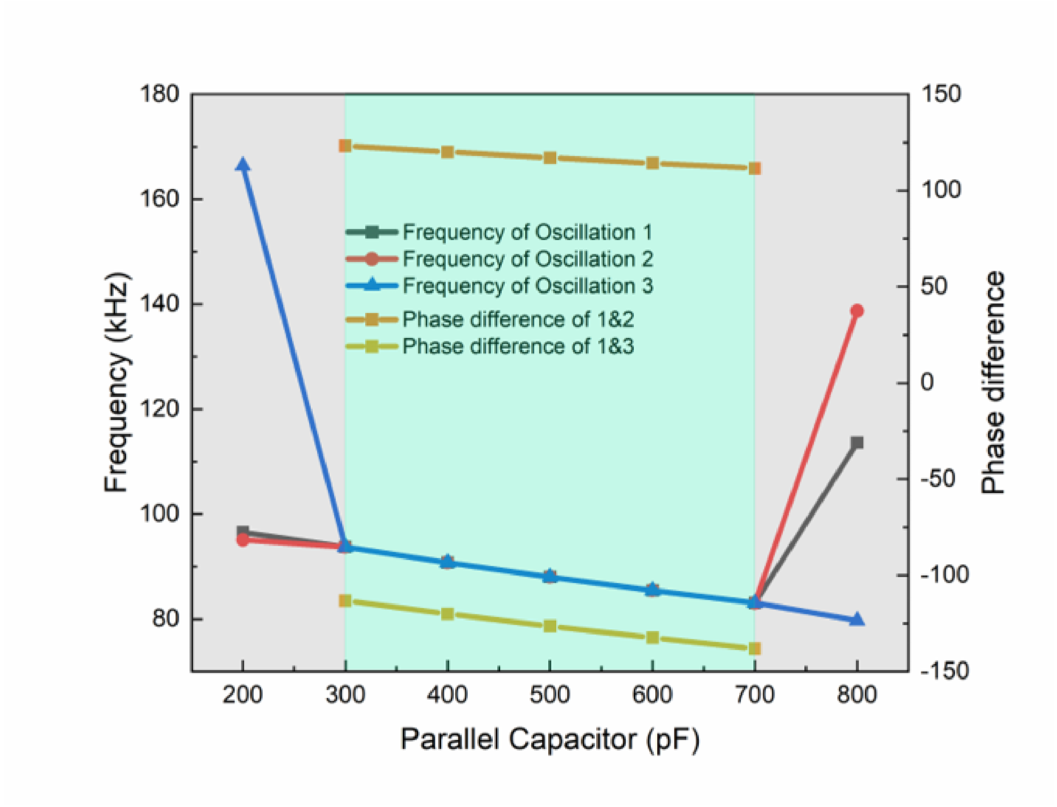

Supplementary Figure 23. Influence of sensing capacitor to three oscillators' coupling.

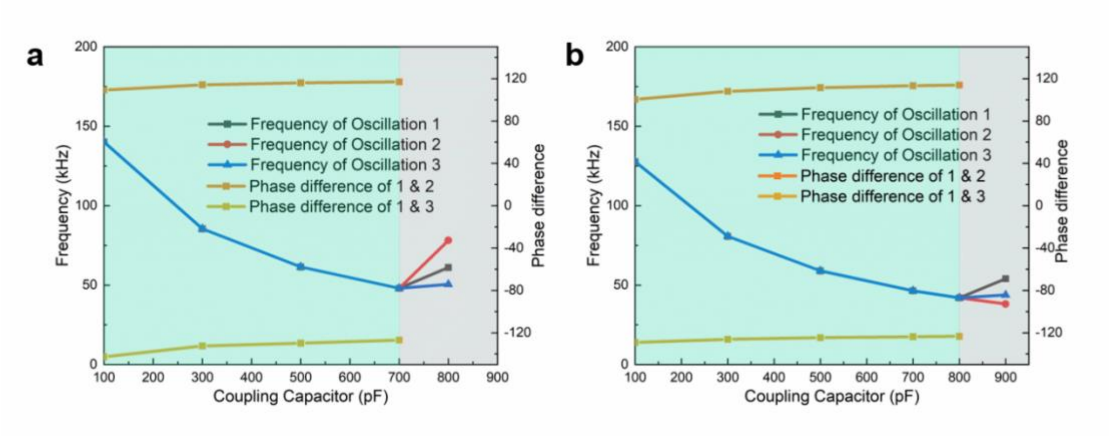

Supplementary Figure 24. Influence of coupling capacitor to three oscillators' coupling in case of mode 001(a) and 011(b).

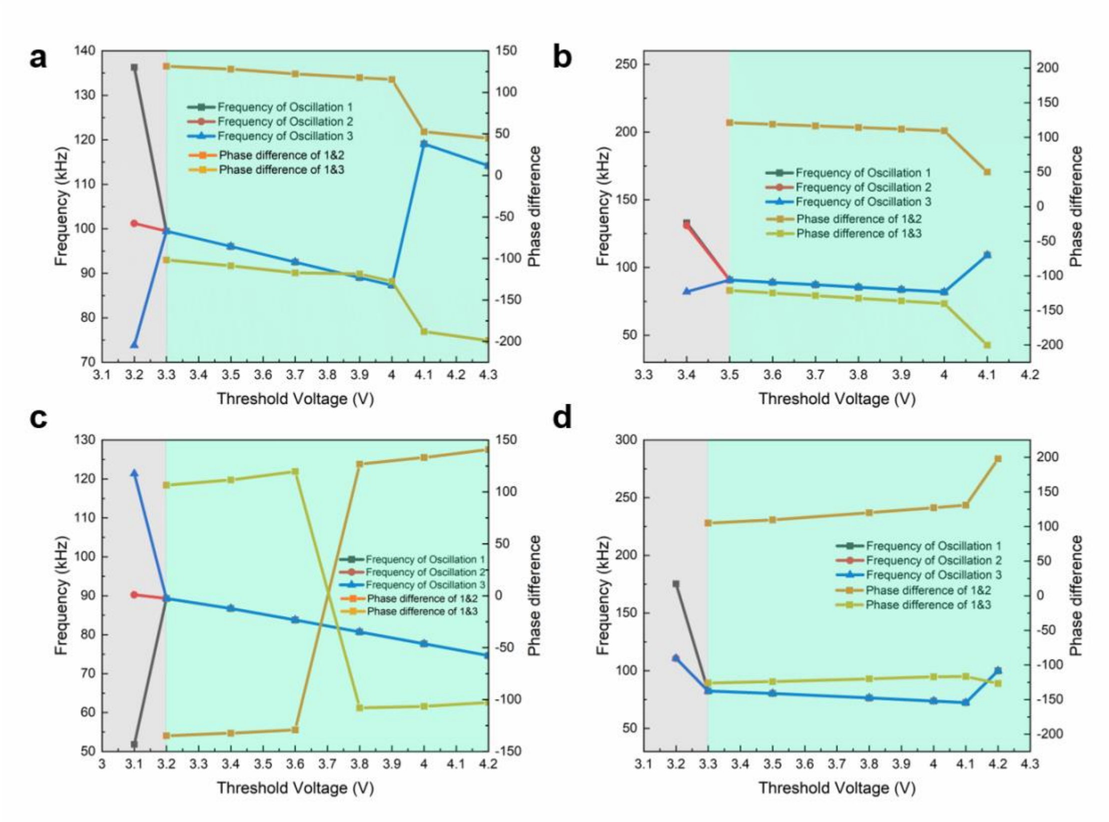

Supplementary Figure 25. Influence of device-to-device variation of V_{th} on three oscillators' coupling in case of mode 000(a), 001(b), 011(c) and 111(d).

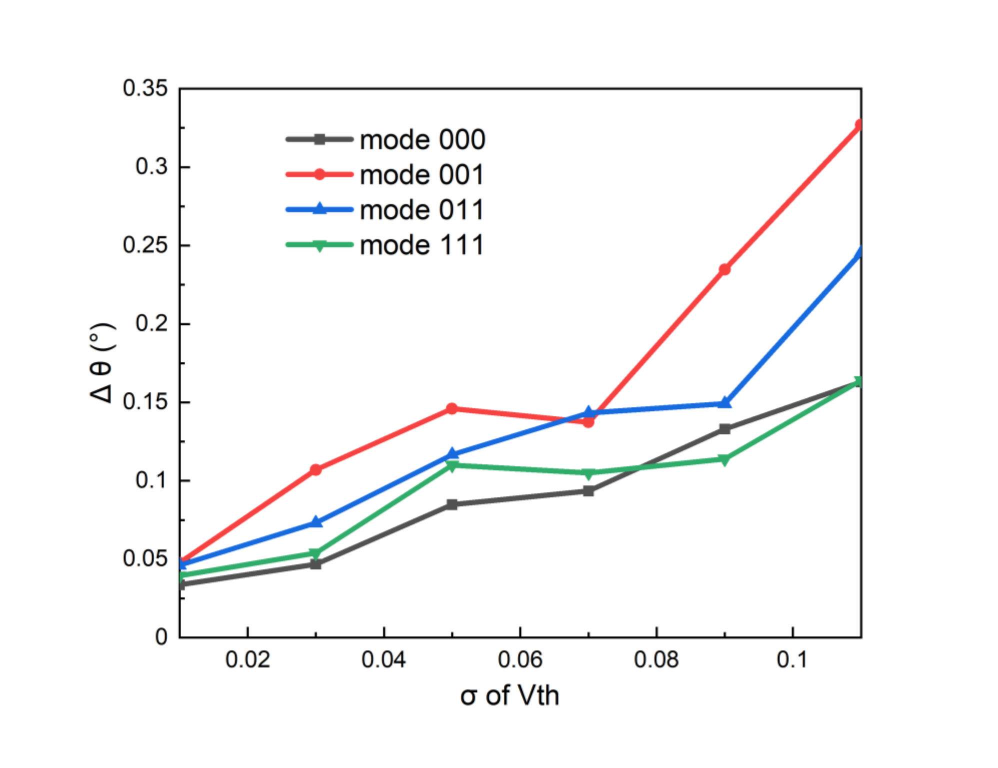

Supplementary Figure 26. Influence of cycle-to-cycle variation of V_{th} on three oscillators' coupling in case of mode 000, 001, 011 and 111.

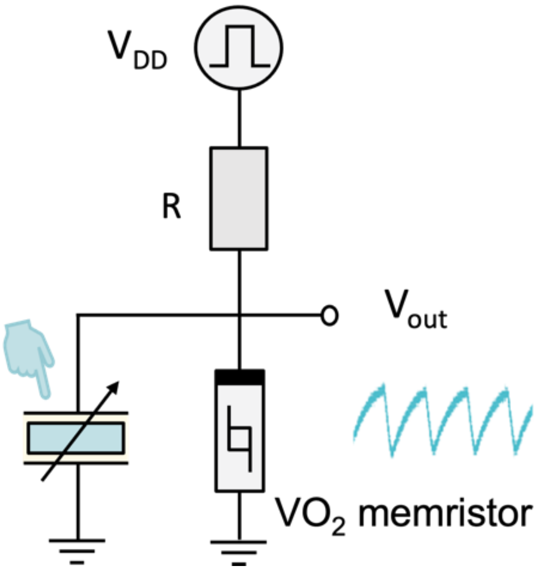

Supplementary Figure 27. The single VO_2 oscillator's circuit.

Supplementary Table 1. The phase mode from experimental ONN to three dynamic sensory neurons.

Input	Phase $difference_{12}$	Phase $difference_{13}$
000	236.38°	104.89°
001	227.59°	71.43°
010	218.51°	120.16°
100	270.47°	126.34°
011	228.67°	88.37°
101	161.66°	252.12°
110	251.28°	139.39°
111	39.00°	165.19°

Supplementary Table 2. A comparison table of gesture recognition task with previous works including memristors and CMOS.

	Zhong, Y. et al.[1]	Chen, B., et al.[2]	Lu L. et al[3]	Ceolini, E. et al.[4]	Rui, Y. et al.[5]	**This work**
**Device**	TiO_x	$MoTe_2$	P3HT/PEO	CMOS	VO_2	VO_2
**Computing style**	Near-sensor computing	In-sensor computing	In-sensor computing	Near-sensor computing	Near-sensor computing	Near-sensor computing
**Computing method**	Reservoir computing	Artificial neural network	Artificial neural network	Spiking neural network	Spiking neural network	**Coupled oscillation**
**Memristor type**	Volatile	Non-volatile	Non-volatile	/	Volatile	Volatile
**D2D variation aware**	NO	NO	NO	/	NO	**YES**
**Device number**	24M+2048M(experiment)	500M (simulation)	5(experiment) *512*128*84 *3(simulation)	/	10R+5C+5M(experiment)	**3R+3M+3C(experiment)** **3 (simulation)**
**Power**	22.2 uW	/	/	29.4 mW	/	7.68 mW
**Time**	1.6 ms	/	/	5.89 ms	/	20 us
**Energy**	13.32 nJ	/	/	173.2 uJ	/	153.6 nJ
**EDP***	21.31 pJ*s	/	/	1.02 uJ*s	/	**3.07 pJ*s**

*The EDP (Energy Delay Product) is the multiply of energy and time, which is the key comparative indicators in neuromorphic computing systems[4].

Supplementary Table 3. The simulation parameter of decision-making network in touch recognition and gesture recognition

Parameters	Touch Recognition	Gesture recognition
J_E	8	4
J_M	-2	-4
I_0	0.8	0.9
α	1.5	1.5
β	4	4
γ	0.1	0.1
θ	6	3
τ_s	1300	200
C	8	0.5

Supplementary Note 1: Environmental variables interact within memristive devices to realize high-order complex behaviors

Computing is essentially a physical process that transforms some inputs into some outputs, and any physical phenomenon can perform a certain kind of computing. We want to build a paradigm of such dynamic computing: by solving a problem (such as the classification problem, the combinatorial optimization problem), while retaining the finite structure of input and output, we can use its continuous time and space capability. Meanwhile, it can interpret the computing results in a limited way and has scalability. The generalized dynamic system can be defined as:

$$\dot{\boldsymbol{x}}(t) = F(\boldsymbol{x}(t))$$

F(t) represents a law of time evolution, *x* represents the state variables in this computing system. This type of system has inherent parallelism and memory characteristics. In a dynamic computing system, time is no longer a counting step of algorithms (no clock required), but a clear physical variable that marks the dynamics of the system. participating in the entire process of computing.

Reflected in electronic circuits, Leon Chua defined the order of memristive devices, which can measure the complexity of dynamic circuits. The equations of the memristive device can be written as:

$$y(t) = g(\boldsymbol{x}, u, t)u(t)$$
$$\frac{d\boldsymbol{x}}{dt} = f(\boldsymbol{x}, u, t)$$

The number of state variable *x* can be defined as the order of memristor.

For the oscillator structure shown in the Figure 2a, it has a second-order memristive complexity. Specifically, the two independent state variables are the internal temperature T of the VO_2 memristive device and the charge Q on the capacitor. When external environment stimuli are applied, changing the paralleling capacitor C. It is equivalent to applying an input to control the oscillator and change its output frequency, while the mode of the dynamic behavior is still oscillation. Therefore, when a single memristive oscillation receives an environmental stimulus and changes its oscillation frequency, it cannot be considered that the oscillator's complexity is increased. Thus, a large number of previously sensor-based single neurons are still summarized in the classical second-order complexity category.

However, when memristive device are coupled together, which means increasing their spatial complexity, things will be different. In this work, we investigate the behavior of sensing stimuli as computational variables in the coupling between nonlinear oscillators, i.e., the interaction of physical elements themselves. In other words, it is transformed into a memory element plus collective behavior. At this point, the environment stimuli can be seen to trigger an additional new element that can increase computational complexity. (If capacitive coupling, it is reflected on Q; if resistive coupling, it is reflected on I). By changing the environment variable to control the dynamic, the system can represent various dynamic behaviors and cause changes in synchronization or not, frequency, phase, and even waveform. This feature demonstrates the dynamic high-order memristive circuit's ability to interact with the

environment and show unique behaviors under different environment stimuli. The new dynamic system is working at the edge of chaos.

Supplementary Note 2: Simulation of the coupled oscillation

To enhance the clarity of the manuscript, especially the computational theory part. Here we supplement the theory and simulation of n coupled oscillators. Assuming that there are n oscillators coupled by capacitance. First, we can extend the formulations presented in Equations 7-9 in the manuscript to encompass more general analytical formulas, as shown below:

$$\mathrm{C_{ii}}\frac{dV_i}{dt}=\frac{\mathrm{V_{dd}}-V_i}{\mathrm{R_0}}-\frac{V_i}{R_{VO_2i}}+\sum_{j\neq i}\mathrm{C_{ij}}\frac{d(V_j-V_i)}{dt}$$

The V_i is the *i*-th oscillator's output. The R_0 is the series resistor. The R_{VO_2i} is the resistor of VO_2 device of *i*-th oscillator. The $\mathrm{C_{ii}}$ is the parallel sensing capacitor of *i*-th oscillator and the $\mathrm{C_{ij}}$ is the coupled capacitor between *i*-th oscillator and *j*-th oscillator.

Note that VO_2 device is a volatile memristor which has four parameters: R_{on}, R_{off}, V_{th} and V_{hold}.

$$if\ V_i>\mathrm{V_{th}}\&\&R_{VO_2i}=\mathrm{R_{off}},R_{VO_2i}\to\mathrm{R_{on}}$$

$$if\ V_i<\mathrm{V_{hold}}\&\&R_{VO_2i}=\mathrm{R_{on}},R_{VO_2i}\to\mathrm{R_{off}}$$

Through transformation, the formula can be written as:

$$\sum_j\mathrm{C_{ij}}\frac{dV_i}{dt}-\sum_{j\neq i}\mathrm{C_{ij}}\frac{dV_j}{dt}=\frac{\mathrm{V_{dd}}-V_i}{\mathrm{R_0}}-\frac{V_i}{R_{VO_2i}}$$

Using the three coupled oscillators discussed in the article as an illustration, when n=3, the formula can be simplified as:

$$\begin{pmatrix} C_{11}+C_{12}+C_{13} & -C_{12} & -C_{13} \\ -C_{21} & C_{21}+C_{22}+C_{23} & -C_{23} \\ -C_{31} & -C_{32} & C_{31}+C_{32}+C_{33} \end{pmatrix} \begin{pmatrix} \dot{V}_1 \\ \dot{V}_2 \\ \dot{V}_3 \end{pmatrix} = \begin{pmatrix} \frac{V_{dd}-V_1}{R_0} - \frac{V_1}{R_{VO_21}} \\ \frac{V_{dd}-V_2}{R_0} - \frac{V_2}{R_{VO_22}} \\ \frac{V_{dd}-V_3}{R_0} - \frac{V_3}{R_{VO_23}} \end{pmatrix}$$

In the application presented in this work, the coupling capacitors value are all same:

$C_{ij} = C_o (i \neq j)$.

Hence, the natural frequency of the *i*-th oscillator can be altered by adjusting C_{ii}.

Solving a coupled system of n oscillators can be accomplished in the following:

$V_i(t=0) = V_{i0}$, $R_{VO_2i} = R_{off}$

for 0 : dt : T

compute $b_i = \frac{V_{dd}-V_i}{R_0} - \frac{V_i}{R_{VO_2i}}$

compute $a_{ii} = \sum_j C_{ij}$, $a_{ij} = -C_{ij}$

$dV = \text{linsolve}(\boldsymbol{b}, \boldsymbol{a}) \times dt$

$\boldsymbol{V}(t+dt) = \boldsymbol{V}(t) + d\boldsymbol{V}$

for i = 1 : n

if $(R_{VO_2i} = R_{off})$&$(V_i(t+dt))$>=V_{th}+C_{var1}

$R_{VO_2i} = R_{on}$;

if $(R_{VO_2i} = R_{on})$&$(V_i(t+dt))$<=V_{hold}+ C_{var2}

$R_{VO_2i} = R_{off}$;

end

end

We simulated the coupling process of 2 and 3 oscillators using MATLAB and investigated the impact of a broad range of parameters. As for the device model, V_{th} = 3.8 V, V_{hold} = 1.6 V, R_{off} = 20 kohm, R_{on} = 300 ohm.

For two-oscillator coupling, We set V_{dd} = 8 V, R_L = 10 kohm，C_o= 300 pF. We set one sensing capacitor is 400 pF, so that the natural frequency of a single oscillator is 402 kHz. Then we change the other oscillator's sensing capacitor to observe the two oscillators' coupling situation. The result is shown in Supplementary Fig 21.
The results depicted in the figure reveal that only when the other oscillator sensing capacitor is in 310 pF- 500 pF, the two oscillators can be synchronized and lock phase difference. The difference in natural frequency is 117 kHz (310pF) and 80kHz (500pF).

When increasing the coupling capacitance, the tolerance of the natural frequency difference will also increase. As shown in Supplementary Fig 22(a), when the coupling capacitance is increased to 500 pF, the natural frequency range is from 310 kHz (510 pF) to 536 kHz (300 pF); when the coupling capacitance is increased to 1 nF, the natural frequency range is from 293 kHz (500 pF) to 555 kHz (290 pF). However, due to the real physical devices having device-to-device variation, the coupling capacitance cannot be too large, otherwise the synchronization will be destroyed. For example, when device 1 and device 2 have different V_{th}, V_{th1} = 3.8V,

V_{th2} = 3.7V, and the coupling capacitance exceeds 1.6 nF, the two devices cannot synchronize as shown in Supplementary Fig 22(b)

For three-oscillator coupling, the parasitic capacitance is set at 200 pF. As shown in Supplementary Fig 23, With the two devices' parallel capacitors fixed at 0.4nF, varying the third capacitance value revealed a valid coupling range in 0.3nF-0.7nF. Furthermore, when two devices' parallel capacitors were set at 0.4nF and one device's parallel capacitor was set at 0.6nF, we modified the coupling capacitor value to observe potential coupling. As illustrated in Supplementary Fig 24 (a) and (b), we observed that coupling persisted until the value increased to 700 pF and 800 pF in case of mode 001 and 011 respectively.

Device-to-device and cycle-to-cycle variability in VO_2 oscillators also have an impact on coupling. Taking the variation of V_{th} as an example, we can configure the coupling state to 000, 001, 011 and 111 mode, setting the V_{th} of two devices simultaneously to 3.8 and manipulating the V_{th} of the third device. The result in Supplementary Fig 25 shows that coupling is unattainable when Vth is below 3.5 at 001 mode, but it remains feasible when Vth exceeds 4.1V at all case. This outcome substantiates that coupling is established within the range of device fluctuations (3.5 < Vth < 4.1V).

Cycle-to-cycle variation will influence the phase resolution post coupling. The phase jitter induced by V_{th} fluctuate during the oscillation represents the lower limit of phase

resolution. As shown in Supplementary Fig 26, we have evaluated the impact of cycle-to-cycle variation in different modes. When the cycle-to-cycle variation increases from 0.01 to 0.11, the maximum value of the phase difference is less than 0.35°. Considering that the cycle-to-cycle variation of the device is within 0.01 (Supplementary Fig 4), The impact of this part is almost negligible.

The variations of other parameters from device to device or cycle to cycle can also be analyzed in a similar way using the formulas and framework we proposed here.

Supplementary Note 3: Energy computing method

The energy consumed mainly in the fire and reset process. The oscillators' circuit is shown in Supplementary Fig 27**:**

It can be described by:

$$\mathrm{C_s}\frac{\mathrm{d}V_{out}}{\mathrm{dt}} = \frac{\mathrm{V_{dd}} - V_{out}}{\mathrm{R_L}} - \frac{V_{out}}{R_{VO_2}}$$

When we measure the output voltage V_{out}, the total current can be computed by:

$$\mathrm{I(t)} = (\mathrm{V_{dd}} - V_{out}(t))/\mathrm{R_L}$$

Then the energy consumption can be computed by:

$$\mathrm{P} = \frac{1}{\mathrm{T}} \times \int_0^T \mathrm{V_{dd}} \times I(t)dt$$

Therefore, the per spike energy can be computed by: $\mathrm{E} = \mathrm{P/frequency}$

When we obtain the oscillation data from experiments, the energy per spike can be computed in this way. The current through the VO_2 is a sharp spike at the falling edge

of voltage oscillation. When the oscillators are coupled, this method is also suitable because the coupling capacitor doesn't cost extra power in a cycle.

Reference:

1. Zhong, Y., et al. A memristor-based analogue reservoir computing system for real-time and power-efficient signal processing. *Nat. Electron.*, **5**, 672-681 (2022).

2. Chen, B., Yao, J., Xia, J., Yang, R., & Miao, X. A Strain-Sensitive Flexible $MoTe_2$-Based Memristor for Gesture Recognition. *IEEE Electron Device Lett.*, ***44***, 622-625 (2023).

3. Liu, L., et al Stretchable neuromorphic transistor that combines multisensing and information processing for epidermal gesture recognition. *ACS nano*, **16**, 2282-2291 (2022).

4. Ceolini, E., et al. Hand-gesture recognition based on EMG and event-based camera sensor fusion: A benchmark in neuromorphic computing. *Front. in Neuro*, **14**, 637 (2020).

5. Yuan, R. et al. A calibratable sensory neuron based on epitaxial VO2 for spike-based neuromorphic multisensory system. *Nat. Commun.*, **13**, 3973 (2022).

6. Dutta, S. et al. Programmable coupled oscillators for synchronized locomotion. *Nat. Commun.*, **10**, 3299. (2019)

7. Kumar, S., Wang, X., Strachan, J. P., Yang, Y., & Lu, W. D.. Dynamical memristors for higher-complexity neuromorphic computing. *Nat. Rev. Mater.*, **7**, 575-591. (2022)

8. Chua, L., Sirakoulis, G. C., & Adamatzky, A. *Handbook of Memristor Networks*. Springer Nature. (2019)

9. Brown, T. D., Kumar, S., & Williams, R. S. Physics-based compact modeling of electro-thermal memristors: Negative differential resistance, local activity, and non-local dynamical bifurcations. *Appl. Phys. Rev.*, ***9***, 011308. (2022)
